# Echocardiography before non-cardiac surgery: current knowledge, guideline recommendations, and clinical evidence – a narrative review

**DOI:** 10.1007/s10877-026-01457-5

**Published:** 2026-06-18

**Authors:** Viktoria Mertin, Alexandra Stroda, Tobias Bruns, Giovanna Lurati Buse

**Affiliations:** Department of Anesthesiology, Medical Faculty, University of Duesseldorf, Duesseldorf, Germany

**Keywords:** Transthoracic echocardiography, Preoperative cardiac testing, Outcome impact, Perioperative management, Guideline adherence, Clinical impact

## Abstract

This narrative review provides an overview of the evidence on transthoracic echocardiography (TTE) at rest in patients undergoing non-cardiac surgery with regard to detection of new diagnoses, TTE-driven management changes, and outcome impact of preoperative TTE. It summarizes the evidence on preoperative TTE and reviews the current recommendations by professional societies from both Europe and Northern America. TTE is a very important non-invasive diagnostic tool to obtain information on cardiac function before surgery. While studies on the effectiveness of preoperative TTE are limited, there is evidence that TTE before non-cardiac surgeries can detect new diagnoses in a relevant proportion of patients. Also, data on changes in management based on TTE findings is scarce. Both the American Heart Association and the American College of Cardiologists and the European Society of Cardiology have recently published updated recommendations regarding the use of TTE before non-cardiac surgery. While both societies are in favour of TTE in symptomatic patients and do not recommend TTE to be performed routinely, several differences remain. Information on outcome benefits in patients having received TTE prior to surgery is not conclusive. In this review we could detect several knowledge gaps concerning the yield and impact on management and outcome of preoperative TTE in non-cardiac surgery patients.

## Background

Postoperative complications represent the third highest leading cause of death globally [1] with cardiovascular complications being the 3rd most common complication after surgery. Thus, preoperative evaluation plays a crucial role in assessing a patient’s risk of postoperative cardiovascular complications. It serves both to inform shared-decision making and to guide perioperative management, e.g., invasive hemodynamic monitoring or postoperative admission to a monitored ward or intensive care unit (ICU). Current guidelines recommend the consideration of the patient’s risk factors and symptoms, of procedural risk, and of cardiac biomarkers or functional capacity to reach an informed decision on additional cardiological evaluation [2–4].

The aim of this narrative review is first, to summarize the evidence on preoperative transthoracic echocardiography (TTE) in patients undergoing non-cardiac surgery with particular focus on its utility to detect new diagnoses, its ability to drive changes in perioperative management, and its impact on perioperative outcomes; second, to review current recommendations by professional societies [3, 4]; and third, to identify knowledge gaps and to propose priorities for future research. This article covers both: comprehensive preoperative TTE and point of care, focused cardiac ultrasound (FOCUS).

## Methodology

A structured PubMed search was performed for English-language publications published between 2010 and 2025 using the following search strategy: (((“echocardiography” OR “preoperative echocardiography” OR “pre-operative echocardiography” OR “focused echocardiography” OR “focused transthoracic echocardiography” OR “TTE” OR “preoperative cardiac testing”) AND (“noncardiac surgery” OR “non-cardiac surgery”)) OR ((“preoperative assessment” OR “cardiovascular assessment” OR “cardiovascular management”) AND (“noncardiac surgery” OR “non-cardiac surgery”)) OR (((“preoperative” OR “pre-operative”) AND (“cardiac testing” OR “preoperative cardiac evaluation” OR “cardiac ultrasound” OR “preoperative cardiac testing”)) OR (“pre-operative echocardiography”))). This search yielded 607 records. Exclusion criteria included case reports, paediatric studies, animal studies, and studies focusing primarily on intraoperative echocardiography and/or transoesophageal echocardiography (TEE). After title and abstract screening, 148 publications with direct relevance to the topic of preoperative transthoracic echocardiographic assessment before non-cardiac surgery (NCS) remained.

The remaining literature consisted predominantly of narrative reviews, guideline documents, guideline summaries, editorials, letters, observational studies, and selected prospective studies relevant to perioperative cardiovascular assessment. Articles were selected based on clinical relevance, and contribution to the understanding of preoperative echocardiographic assessment in NCS. Screening and selection were performed by two authors, with disagreements resolved by discussion and consensus. In addition to the database search, relevant guideline documents and key reference publications identified through manual screening of references and expert consensus were included if considered essential for the clinical and methodological context of perioperative echocardiographic assessment.

## Contribution of TTE and FOCUS for the detection of new diagnoses/findings

Transthoracic echocardiography at rest is the main diagnostic tool to non-invasively obtain information crucial for intra and perioperative management like systolic and diastolic left- and right-ventricular function, valvular integrity and function, and signs suggestive of pulmonary hypertension [5]. During a comprehensive TTE, a systematic assessment is performed, which however requires not only trained personnel but also sufficient institutional capacity, making it a limited resource. A FOCUS examination is a targeted, problem-oriented assessment designed to answer specific clinical questions. According to the current ESC guidelines [3], FOCUS examinations are a valid alternative to an extensive TTE examination in the echocardiography lab (Class IIb recommendation) to obtain information without delaying surgery.

Evidence regarding the contribution of echocardiography for the detection of new diagnoses has predominantly been generated in the non-elective surgery setting. Also, most studies cited here have used FOCUS examinations, probably because of the abovementioned reasons. In a systematic review, Heiberg at al. summarized the evidence on the detection of previously unknown pathologies by FOCUS before non-elective NCS. In the 6 included studies (total *n* = 607 patients), the frequency of new diagnoses ranged from 17% to 78%. The most frequent new diagnoses were left-ventricular dysfunction (in 0–23% of patients), valvular pathologies (in 1–48% of patients), and hypovolemia (in 1–34% of patients) [6]. In a more recent study (*n* = 163), Pallesen et al. reported the detection of new information by FOCUS in 45% of patients planned for non-elective orthopaedic or abdominal surgery [7]. Again, the new findings pertained most frequently to left ventricular systolic function (20%), aortic valve pathology (20%), and volume status (20%). Among 99 patients requiring urgent NCS, FOCUS excluded major pathologies in 25% of patients with suspected cardiac disease (while confirming the clinical suspicion of significant pathologies in the remaining patients). Among patients without suspected cardiac disease, it detected previously unknown and unsuspected relevant findings in 29% of patients [8]. In a small but more recent sample of patients undergoing various non-cardiac urgent procedures(*n* = 57), FOCUS excluded relevant pathologies in 19% of patients with suspected cardiopulmonary disease and corroborated the clinical suspicion in the remaining patients, e.g., by detecting valvular pathologies (31%), right ventricular systolic failure (12.5%), LV systolic failure (56%) and LV diastolic failure (12.5%) (some patients had more than one pathology). Further, it detected new relevant findings in 68% of patients without suspected cardiopulmonary disease. Therefore, FOCUS changed the classification as cardiopulmonary diseased or not in more than half of the patients [9].

To summarize, especially FOCUS prior to NCS appears to detect new diagnoses in a relevant proportion of patients. It also seems to contribute to “downgrading” a considerable proportion of patients in whom cardiac disease is clinically suspected. However, this evidence is limited in term of quantity (number of studies) and of certainty (mostly single-centre design, limited number of patients, selected populations, lack of information on classification as clinically suspected cardiovascular disease).

## Examination-driven management changes

The detection of new diagnoses or exclusion of relevant pathologies is expected to result in changes in the perioperative management, e.g., a step-up in hemodynamic monitoring, choice of anaesthetic technique, or admission to an intensive care or high-dependency unit. The seven studies (total *n* = 1068) included in a systematic review by Heiberg et al. reported changes in the perioperative management ranging from 12 to 82% of patients after FOCUS. These findings led to changes in the medical management in 4–52%, in a different anaesthesia technique in 3–52%, and in a modification of postoperative disposition in 6–34% of patients [6]. Other actions triggered by FOCUS findings were adaptation in volume management and referral for further examination, more rarely surgery postponement or even cancellations [9].

To summarize, while available evidence suggests FOCUS-triggered management changes in a conspicuous proportion of patients, the data are scarce, of limited certainty, and frequently generated more than a decade ago.

## Impact of preoperative TTE for perioperative outcomes

Wijeysundera et al. evaluated the association between TTE at rest in the 6 months before intermediate or high-risk NCS and 30-day and 1-year survival in a large administrative database. Among over 35,000 propensity-score matched pairs, the relative risk was 1.14 (95% confidence interval [CI] 1.02 to 1.27) for 30-day mortality and 1.07, (95%CI 1.01 to 1.12) for 1 year mortality [10], favouring non-TTE patients. Similarly, among US Veterans with known coronary artery disease (*n* = 26,641), preoperative TTE at rest was independently associated with 30-day MACE (adjusted odds ratio 1.92, 95% CI 1.66 to 2.2) [11]. An adverse association of preoperative TTE for 90-day mortality, 1-year mortality, length of stay, and 1-year costs was also described for patients with hip fracture (2,298 matched pairs) in another North American administrative database [12]. While residual confounding appears the more probable explanation for these surprising findings, harm, e.g., from unwarranted downstream coronary interventions or drug initiations, cannot be excluded.

The evidence from randomized studies on the impact of echocardiography for perioperative outcomes is very limited. Pallesen et al. randomized 327 patients aged ≥ 65 years undergoing orthopaedic or abdominal non-elective procedures to preoperative FOCUS vs. no FOCUS. The primary endpoint, a composite of 30-day death or prolonged length of stay > 10 days did not differ between the FOCUS and the control group. The same applied to all other endpoints assessed, except for a difference of + 0.23 days (95% 0.04–0.42) longer in-hospital stay within the first 90 postoperative days in the FOCUS group [7]. Among 102 patients with hip fracture randomized to preoperative FOCUS vs. no FOCUS, neither 30-day outcome [13] not 1-year mortality [14] differed between groups.

Several studies [15–18] addressed a slightly different question, i.e., in how far guideline-adherent conduction of TTE (overuse vs. underuse vs. guideline adherent use) affects outcome. Of note, this question is rather an indirect evaluation of the accuracy of selection criteria suggested for TTE conduction by the various guidelines than an assessment of the outcome impact of TTE itself. As such, those studies are discussed below.

To summarize, evidence on the outcome impact of the conduction of TTE before surgery relies largely on administrative data [10–12] and appears affected by residual confounding. The few randomized trials were non-conclusive and underpowered [7, 13, 14].

## Current recommendations for preoperative TTE

Guidelines to assist clinicians in assessing the requirement of a TTE at rest before surgery for individual patients were issued by the European Society of Cardiology (ESC) [3] in 2022 and jointly by the American Heart Association (AHA) and the American College of Cardiologists (ACC) in 2024 [4].

In the 2022 ESC guideline, the indication for preoperative TTE was broadened compared to the version from 2014. The ESC guidelines differentiate several situations as summarized in Table 1. In asymptomatic patients ≥ 65 years of age or patients with cardiovascular risk factors or asymptomatic patients with established cardiovascular disease TTE is not recommended for low-risk procedures, whereas the guideline gives an IIb recommendation for intermediate NCS with poor functional capacity or high NT-proBNP/BNP and a class I recommendation for high-Risk NCS with poor functional capacity or high NT-proBNP/BNP. A class I recommendation is also given for symptomatic patients, patients with a history of genetic cardiomyopathy or newly detected murmurs. For new murmurs, they give a class I recommendation for high-risk NCS and a class IIa recommendation for intermediate risk NCS. Also, a class IIa recommendation is given for patients with suspected new cardiovascular disease or unexplained signs or symptoms. Regarding FOCUS echocardiography, the guidelines states that a FOCUS exam may be performed by trained personnel to avoid delaying surgery (Class IIb). Further, although cardiac auscultation is limited, evidence is insufficient to support replacing it with routine pre-operative FOCUS [3].

**Table 1:** Overview of the guidelines’ recommendations [3]

Clinical Scenario / Indication	Estimated surgical risk	ESC 2022
*Symptomatic patients* (murmurs, chest pain, dyspnoea, or peripheral oedema)	Any risk	Class I
*Newly detected murmur*,* i.e. without symptoms or clinical signs of CVD*	Intermediate-risk NCS	Class IIa
	High-risk NCS	Class I
Family history of genetic cardiomyopathy	Regardless of symptoms and age	Class I
S*uspected new CVD or unexplained signs or symptoms*	High-risk NCS	Class IIa
*Asymptomatic patients < 65 years without any CVD/ cardiovascular risk factors* (such as hypertension, smoking, dyslipidaemia, diabetes, family history of CVD)	Low and intermediate-risk NCS	Class III
	High-risk NCS + poor functional capacity or high NT-proBNP/BNP	Class IIb
*Asymptomatic patients ≥ 65 years of age or patients with cardiovascular risk factors or asymptomatic patients with established CVD*	Low-risk NCS	Not recommended
	Intermediate NCS with poor functional capacity or high NT-proBNP/BNP	Class IIb
	High-Risk NCS with poor functional capacity or high NT-proBNP/BNP	Class I

The AHA/ACC guideline does not apply a matrix based on age, the presence of cardiovascular risk factors and procedural risk. Instead, the authors advocate to estimate the risk in *asymptomatic* patients based on risk calculators and modifying factors, e.g. severe valvular heart disease/ pulmonary hypertension, elevated-risk, congenital heart disease, coronary stents/coronoary artery bypass graft, stroke, pacemaker/ Implantable Cardioverter Defibrillator), and frailty. In case of elevated risk or in presence of modifying factors, the algorithm suggests the measurement of NTproBNP (Class IIa) or troponin (Class IIb) in case of limited functional capacity and upon multidisciplinary discussion, further cardiac exams, among other TTE [4]. For patients with new dyspnoea, physical examination findings indicating heart failure, or new/worsening ventricular dysfunction, it is recommended to perform a pre-operative evaluation of the left ventricular function before undergoing NCS (Class I). In patients with known or suspected severe valvular disease (e.g., aortic stenosis or mitral regurgitation), a TTE is also recommended prior to NCS (Class I). In patients undergoing NCS with a known diagnosis of HF with worsening dyspnoea or any other change in clinical status, pre-operative assessment of left ventricular function is classified as reasonable (Class IIa). The recommendation by the AHA/ACC are summarized in Table 2.

**Table 2:** Overview of the guidelines’ recommendations [3, 4].

Clinical Scenario / Indication	Estimated surgical Risk	2024 AHA/ACC
Known LV dysfunction, clinically stable, no change in status	Low risk (< 1%)	Class IIb
Asymptomatic, clinically stable patients without known cardiovascular disease		Class III
Suspected moderate or severe valvular disease (e.g., murmur or symptom change)	Elevated risk (≥ 1%)	Class I
Dyspnoea of unknown origin		Class IIa
Known heart failure with worsening dyspnoea or change in clinical status		Class IIa
Any suspected structural heart disease based on symptoms or physical exam like dyspnoea	Any risk	Class I
Previously documented LV dysfunction without clinical change, and no TTE in > 12 months		Class IIb
Routine evaluation of LV function in asymptomatic, clinically stable patients		Class III

## Evidence on the adequacy of criteria for TTE conduction in the guidelines and guideline adherence

The impact of preoperative TTE and FOCUS on outcome remains uncertain. The impact of guideline-adherent TTE use vs. under- or overuse remains also unclear. In an administrative database including > 230,000 patients, appropriate TTE compared to non-appropriate TTE was not associated with a reduction of major adverse cardiac events [16]. In a secondary analysis of a prospective cohort (*n* = 6959), neither over- nor underuse of TTE affected adverse cardiac events at 30 days compared to guideline-adherent TTE conduction [15]. It is noteworthy that both analyses were based on earlier guideline versions.

The 2022 updated ESC guidelines expanded the population in whom preoperative TTE may be considered (Class IIb), which may pose a logistical challenge for most institutions. In a 1-day cross-sectional study in a French tertiary centre, 17% of patients planned for NCS fulfilled criteria to consider preoperative TTE [19]. In a secondary analysis of a prospective cohort with over 15,000 patients, the 2022 recommendation did not appear to improve accuracy with regard to patient selection for TTE [20] compared to the earlier 2014 version: the proportion of patients with EF < 40% receiving a stronger recommendation for TTE conduction was 15%, whereas 26% received a weaker recommendation for TTE (net improved recommendation −11% (95%CI −17% to −6%)). Among patients with EF ≥ 40%, 36% received a stronger TTE recommendation and only 10% a weaker one (net improved recommendation − 26% (95%CI −28 to −25%) [20]. Further, guideline-adherent use of TTE was not associated with improved outcomes.

The adherence to guideline-recommendations regarding TTE conduction appears modest. In the aforementioned French study only 21% of the recommended TTEs were actually performed, likely reflecting limited capacity and logistical constraints [19]. Adherence was even lower when B-type natriuretic peptide criteria were applied: 0/60 patients fulfilling the criteria for TTE based on B-type natriuretic peptide received a preoperative TTE [19]. Among patients with chronic stable heart failure (*n* = 3380), only 43% (476/1101) and 32% (756/2362) were submitted to TTE in the 6 months prior to high-risk and intermediate risk surgery, respectively [21]. While limited availability appears a plausible reason for the limited adherence to TTE guideline recommendations, there are only few studies exploring barriers and facilitators for TTE- guideline-adherence. Among over 15,000 European patients, TTE overuse (performed despite class III criteria) and underuse (not performed despite class I criteria) was detected in 16% and 6% of patients, respectively. Of note, geographical region played a relevant role in the level of adherence, with Northern Europe having the highest adherence [18]. Of note, the study reported on adherence to the 2014 guidelines and data for the 2022 ESC guidelines are lacking.

To summarize, while guidelines are available to guide the use of TTE prior to NCS, the criteria used to define which patients should be submitted to TTE are not fully congruent among different professional societies. Further, the accuracy of the criteria, at least those proposed by the ESC in 2022, appears questionable as the updated recommendations led to a large number of TTE without increased case detections. Finally, TTE recommendations do not appear to be systematically translated into clinical practice. While availability seems a plausible factor, reasons for nonadherence to the guidelines are underexplored.

## Knowledge gaps, ongoing studies, and future research priorities

We detect the following knowledge gaps in the current evidence on TTE and FOCUS, especially bearing in mind that studies differ greatly with regard to the use of TTE and FOCUS before NCS, limiting comparability and making definite conclusions difficult.

1. *Patient selection for TTE before NCS*: Current guideline criteria for patient selection do not appear efficient, at least regarding severe systolic LV dysfunction. Information on other major pathologies, e.g. right ventricular function is not available. The EuPreCHO study (https://esaic.org/study/euprecho/, NCT06409234) is a prospective observational study sponsored by the European Society of Anaesthesiology and Intensive Care aiming to establish criteria for patient selection that maximizes the yield of severe pathologies, e.g., severe left and right ventricular dysfunction, severe left-side valvulopathy, diastolic dysfunction) in patients planned for NCS.
2. *Diagnostic yield and clinical management impact*: FOCUS prior to NCS appears to detect new diagnoses. Data from single centre studies of limited size suggest management changes in a relevant proportion of patients of specific patient populations, the certainty and quality of this evidence is so limited however that it cannot be considered conclusive. The EuPreCHO study will prospectively collect information on current practices and management decisions after preoperative TTE in European centres.
3. *Outcome impact*: Data on the outcome impact of TTE arise from administrative data and suffers from residual confounding. The EUPreCHO study will collect disability (using the 12-item WHODAS 2) in 5000 patients submitted to TTE and 2500 not submitted to TTE within the last 6 months before intermediate or high-risk procedure. It may therefore provide some insights on the outcome impact. However, due to the observational design, in spite of advanced statistical techniques, the evidence might also suffer from residual confounding. Moreover, the ECHONOF III trial is expected to provide an answer on the impact of systemic TTE in patients with femoral neck fracture [22]. This ongoing multicentre randomized controlled trial allocates patients to systematic vs. no systematic FOCUS before emergency surgery for femoral neck fractures. The planned sample site is 2000 patients. Endpoints include all-cause mortality, quality of recovery and of life, and postoperative complications [22].
4. *Barriers and facilitators to guideline-adherent TTE conduction prior to NCS*: While some “epidemiological” data describing factors associated with non-adherence to TTE guidelines is available, mixed-methods studies and qualitative research is needed to elucidate factors resulting in limited guideline-adherence and to design education intervention or knowledge transfer intervention to improve it. However, before it is possible to transfer knowledge this has to be generated.

**Fig. 1:**
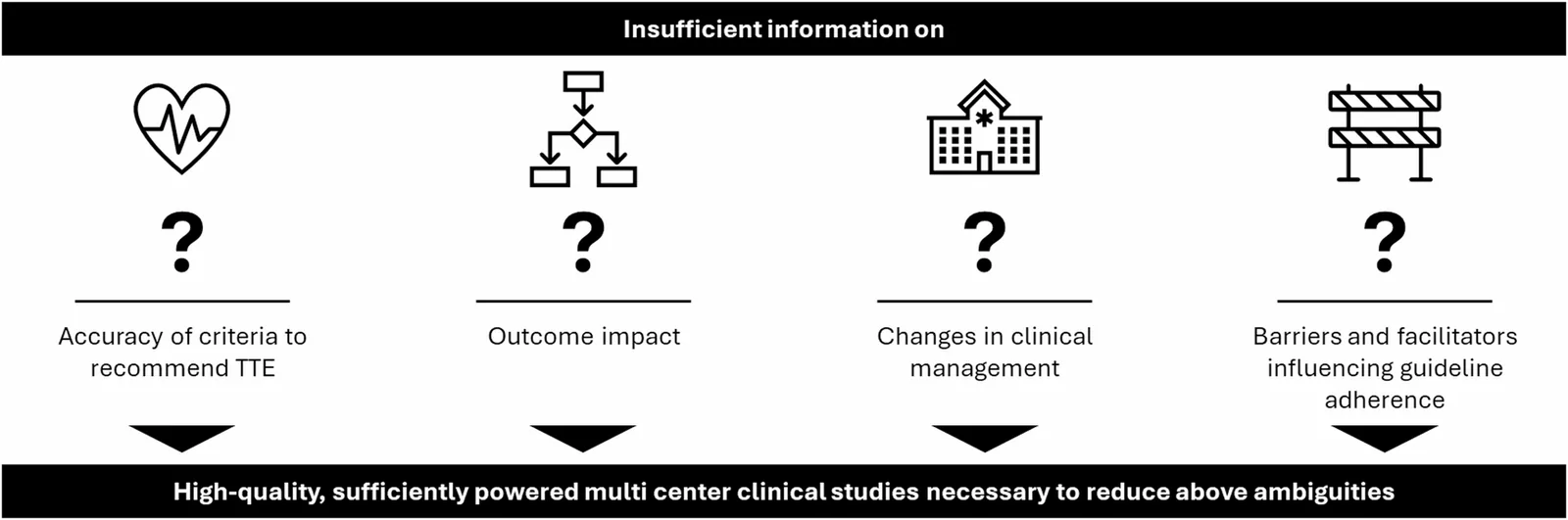
Current state of knowledge

The abovementioned can be visualised in Fig. 1, stressing that the current lack of knowledge in different fields should be addressed by large and high-quality studies in the future.

## Conclusion

To conclude, we described several major knowledge gaps regarding TTE and FOCUS use before NCS. Generally, preoperative echocardiography should not be used routinely, but selectively in clearly defined clinical scenarios, while robust evidence for improved outcomes remains limited. While some of these gaps are being addressed by ongoing studies, more data need to be generated. This specifically concerns patient selection for an efficient and cost-effective use of limited TTE resources, the outcome impact of TTE, and barriers to adequate TTE use (including education). Key aim in all studies should be to design interventions to reach evidence-based TTE utilization with the final aim of improving outcomes in patients with suspected cardiovascular disease submitted to NCS.

## Data Availability

No datasets were generated or analysed during the current study.

## Abbreviations

ACC: American College of Cardiology
AHA: American Heart Association
BNP: B-type natriuretic peptide
COR: Class of recommendation
CI: Confidence interval
CVD: Cardiovascular diseases
ECG: Electrocardiography
ESC: European Society of Cardiology
EuPreCHO: European study on perioperative management and outcome following Preoperative Transthoracic Echocardiography in non-cardiac surgery patients
FOCUS: Focused cardiac ultrasound
HF: Heart failure
ICU: Intensive care unit
LV: Left ventricle
NCS: Non-cardiac surgery
NT-proBNP: N-terminal pro–B-type natriuretic peptide
TTE: Transthoracic echocardiography
